# Artificial medial and lateral meniscus prostheses mimic kinematics of the native meniscus without affecting knee joint motion: A cadaveric study

**DOI:** 10.1002/jeo2.70379

**Published:** 2025-07-24

**Authors:** Branco S. van Minnen, Alexis R. Sturm, Albert J. van der Veen, Sebastiaan A. W. van de Groes, Nico Verdonschot, Tony G. van Tienen

**Affiliations:** ^1^ Orthopaedic Research Lab, Radboud University Medical Centre Nijmegen The Netherlands; ^2^ ATRO Medical B.V. Uden The Netherlands; ^3^ University of Florida Gainesville Florida USA; ^4^ Department of Orthopaedics Radboud University Medical Centre Nijmegen The Netherlands; ^5^ Laboratory for Biomechanical Engineering University of Twente Enschede The Netherlands

**Keywords:** biomechanics, knee joint, knee kinematics, meniscectomy, meniscus prosthesis

## Abstract

**Purpose:**

This exploratory study tested the hypothesis that total medial and lateral meniscus prostheses have no adverse effect on range of motion, kinematics and laxity of the knee joint. Furthermore, it was investigated whether the prosthesis kinematics were similar to the native meniscus kinematics during flexion and under knee loading.

**Methods:**

A dedicated knee testing rig was used to apply different flexion angles and joint loads to 13 cadaveric knee joints. Roentgen stereophotogrammetric analysis (RSA) was used to analyse the kinematics of the knee joint and the meniscus. For both the medial and lateral compartment, linear mixed models were used to make a comparison between the native condition, the meniscectomized knee joint, a meniscal allograft transplant and the meniscus prosthesis. A Lachman test was simulated to measure the effect of the different meniscal conditions on anteroposterior knee laxity, with and without the anterior cruciate ligament intact.

**Results:**

None of the meniscal conditions restricted range of motion or adversely affected joint kinematics. During flexion and loading, the medial and lateral meniscus prostheses translated over the tibial plateau in a comparable way as the native meniscus, although some differences were identified. Anteroposterior knee laxity was not significantly altered by any of the meniscal conditions.

**Conclusions:**

The artificial medial and lateral meniscus prostheses did not affect knee range of motion. Only minor differences in knee joint and meniscus kinematics were found between the prostheses and the native meniscus, and no effect on joint laxity was found.

**Level of Evidence:**

Level V.

**Abbreviations**
ACL: anterior cruciate ligament
AP: antero‐posterior
MAT: meniscal allograft transplantation
ML: medio‐lateral
OA: osteoarthritis
PCU: polycarbonate urethane
PD: proximo‐distal
PEEK: polyether ether ketone
PET: polyethylene terephthalate
PMMA: polymethyl methacrylate
RoM: range of motion
RSA: Roentgen stereophotogrammetric analysis

## INTRODUCTION

The medial and lateral meniscus play a vital role in biomechanics and lubrication of the knee joint [9, 13, 18]. The medial meniscus also has a stabilising function, especially in ACL‐deficient knees [9]. Meniscal tears can affect joint mechanics and cause pain, which still commonly results in surgical removal of (part of) the meniscus [1, 11]. However, meniscectomy may cause the contact stresses in the cartilage to increase significantly, induce progressive knee pain and increase the risk of developing osteoarthritis (OA) [3, 15, 17, 18].

For patients suffering from post‐meniscectomy pain, some solutions are currently available. After partial meniscectomy, implantation of a meniscal scaffold may result in favourable outcomes [19, 24]. Meniscal allograft transplantation (MAT) has proven to be a feasible solution to relieve pain after total medial or lateral meniscectomy, but the availability is limited [14, 22, 28]. Artificial total meniscus prostheses may therefore be an attractive alternative to replicate the function of the meniscus and to reduce knee pain [6, 24]. During a first‐in‐human study with an anatomically shaped medial meniscus prosthesis, patients experienced persistent pain and limited range of motion (RoM) [23]. The main mechanisms behind the RoM deficit were found to be related to the flexibility of the prosthesis and its fixation. In general, the prosthesis did not sufficiently extrude out of the joint under loading and was unable to follow the displacements of the tibiofemoral contact point. Preventing direct cartilage contact between femur condyle and tibia plateau resulted in impingement of the anterior horn in extension. During flexion, the femur climbed on top of the posterior prosthesis horn, eventually limiting deep flexion.

Rigorous design changes have made the medial meniscus prosthesis more flexible and added a fixation technique allowing translation over the tibial plateau [16]. Previous studies demonstrate its potential to partially restore tibiofemoral contact mechanics after meniscectomy [16]. Recently, the learnings from the medial meniscus prosthesis have been implemented in the design of the first anatomically shaped lateral meniscus prosthesis.

The first objective of this study was to confirm that implantation of the medial or lateral meniscus prosthesis does not adversely affects the knee joint RoM. Second, the phenomenon of the femur climbing on top of the prosthesis during flexion of the knee was investigated by measuring joint kinematics. Third, extrusion of the prostheses was evaluated by comparing its kinematics with the native meniscus and meniscal allograft, during flexion and under loading. Finally, a Lachman test was performed to assess potential effects of the meniscus prosthesis on the anteroposterior laxity of the knee.

It was hypothesised that the current version of the meniscus prosthesis do not affect RoM or joint kinematics, as it is expected to move similarly to the native meniscus and meniscal allograft. For the same reason, no major effect on knee laxity was expected.

## MATERIALS AND METHODS

**Figure 1:**
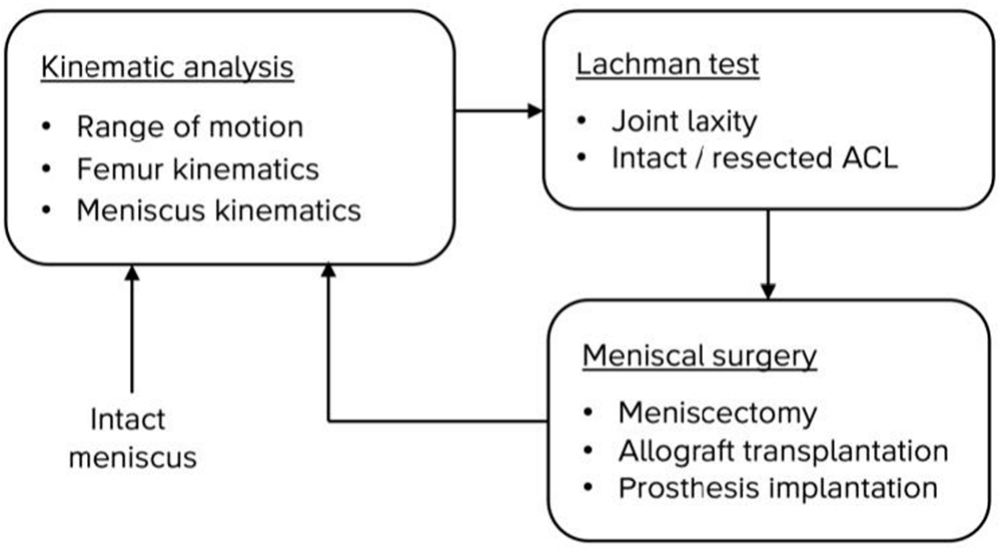
Infographic summarising the test protocol. ACL, anterior cruciate ligament.

**Figure 2:**
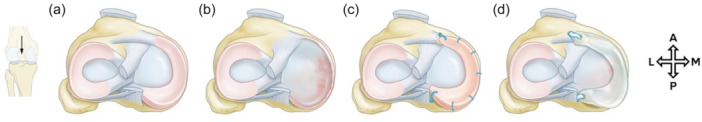
Schematic overview of the different medial meniscal conditions in a left knee. The same conditions were tested for the lateral meniscus. (a) Native meniscus. (b) Medial meniscectomy. (c) Meniscal allograft. (d) Meniscus prosthesis. The arrows indicate the anterior (A), posterior (P), lateral (L) and medial M) sides of the tibial plateau.

This cadaveric study consisted of experiments with both the medial and lateral meniscus prosthesis in two different experimental setups, as summarised in Figure 1. First, the kinematics of the knee joint (i.e., movement of the femur relative to the tibia) and the meniscus prosthesis (i.e., movement of the meniscus relative to the tibia) were evaluated in a joint simulator, using Roentgen stereophotogrammetric analysis (RSA). RSA enables tracking of tantalum beads inside the femur, tibia and meniscus with an accuracy of 50 µm [5, 26]. A comparison was made between the intact native meniscus, total meniscectomy, meniscal allograft, and the meniscus prosthesis (Figure 2). Second, a Lachman test was simulated to assess anteroposterior joint laxity. This test was also performed with the anterior cruciate ligament (ACL) resected, to potentially magnify the effect of the different meniscal states. The sequence of testing the different meniscal states was alternated as much as possible, to minimise the effect of degradation of the cadaveric specimens.

### Cadaveric specimens

After ethical approval was obtained from the anatomy departments of the VU University Medical Center (Amsterdam, the Netherlands) and the Radboud University Medical Center (Nijmegen, the Netherlands), 13 fresh‐frozen human cadaveric knee joints were obtained. Seven specimens were used to test the effects of different meniscal conditions on the medial side and six were used for the lateral side. Specimens were not used if the compartment of investigation was significantly osteoarthritic, had severe meniscal damage, or showed evidence of prior knee surgery.

### Artificial meniscus prosthesis

**Figure 3:**
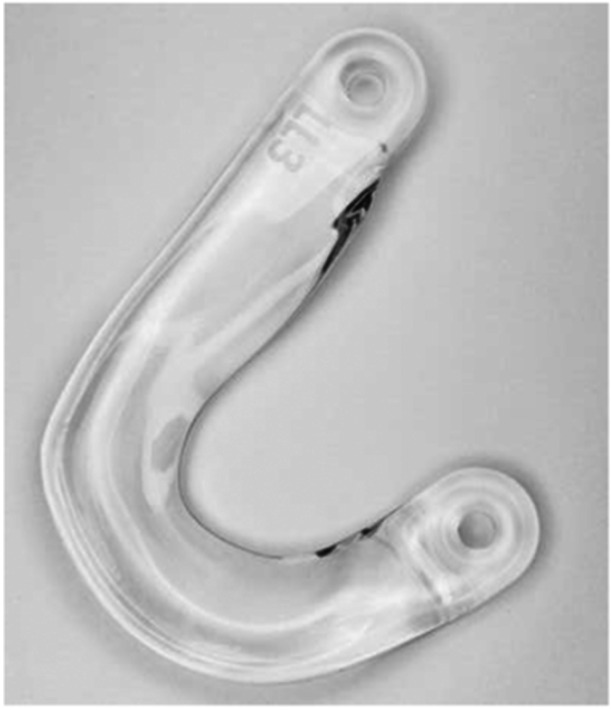
Artimis® lateral meniscus prosthesis.

**Figure 4:**
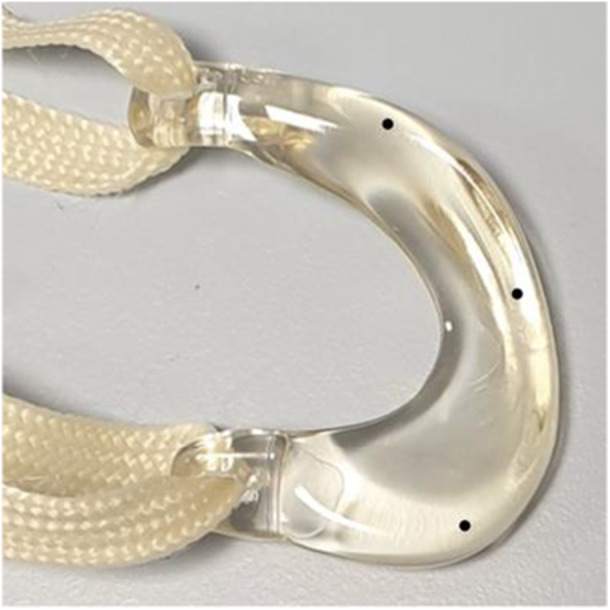
Average‐sized, left medial meniscus prosthesis, including fixation tape and tantalum bead positions.

The Artimis® meniscus prosthesis (ATRO Medical, Nijmegen, The Netherlands) is based on the material properties and geometry of a healthy meniscus [25]. It is a permanent implant, intended to replace the meniscus in patients who experience pain following total meniscectomy. The lateral (Figure 3) and medial (Figure 4) meniscus prostheses are available in five different sizes for both the left and right knee. The main body of the prosthesis is made of the flexible Bionate® II 80A polycarbonate urethane (PCU), which is connected to two horn attachments made of the stronger Bionate® 75D. The meniscus prosthesis is fixated to the tibial plateau using a polyethylene terephthalate (PET) fixation tape, which is secured in bone tunnels by polyether ether ketone (PEEK) interference screws.

### Surgical technique

The cadaveric specimens were prepared by removing all excess skin, fat and muscle, ensuring the collateral and cruciate ligaments are left intact. The tibia and femur were cut to size to fit in the testing device and secured in pots using PMMA bone cement. In this study, a comparison was made of the native knee, after meniscectomy, meniscal allograft transplantation, and implantation of the meniscus prosthesis. Every knee was first tested with the native meniscus intact, after which a total medial or lateral meniscectomy was performed. For the prosthesis implantation and allograft transplantation, an aiming device was used to drill two tunnels through the tibia. The fixation tapes shown in Figure 4 were pulled into the bone tunnels and secured with interference screws.

**Figure 5:**
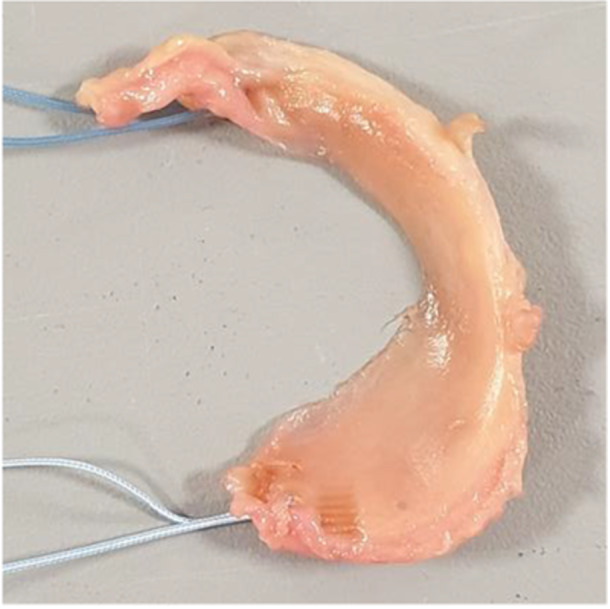
Native medial meniscus, excised from a cadaveric knee joint and prepared for use as an ‘allograft’.

Figure 5 shows how the excised native meniscus was prepared to mimic an allograft by attaching sutures to both meniscal horns. If the autograft was of insufficient quality, a size‐matched excised meniscus of a different specimen was used. The allograft was fixed to the tibial plateau using the same bone tunnels and interference screws. Capsular fixation of the allograft was performed only if it was deemed necessary by the orthopaedic surgeon to avoid ‘bucket‐handle’‐like dislocation. The lack of capsular fixation was not expected to affect meniscus kinematics [27].

After tests were completed with the four different meniscus conditions, an ACL resection was performed. Subsequently, the Lachman test was performed again in the meniscectomized knee and after implantation of the prosthesis and the allograft, in alternating order.

### Kinematic study

**Figure 6:**
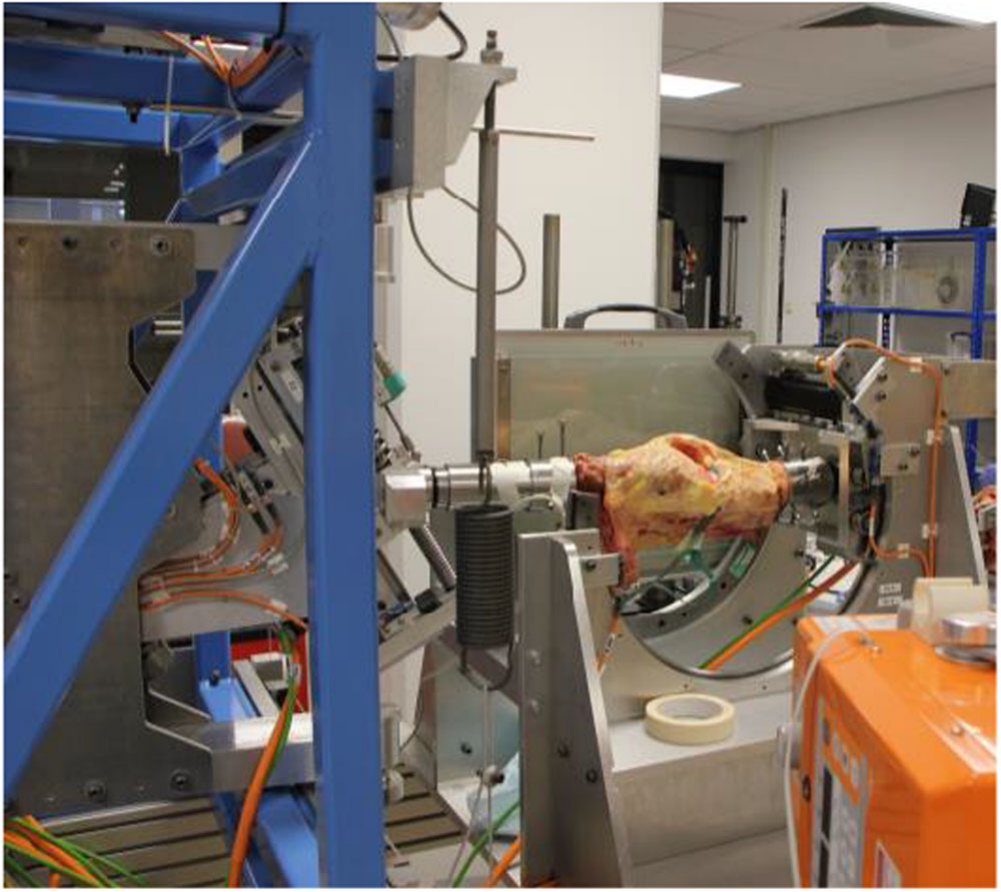
Picture of the test setup for the kinematic study.

**Figure 7:**
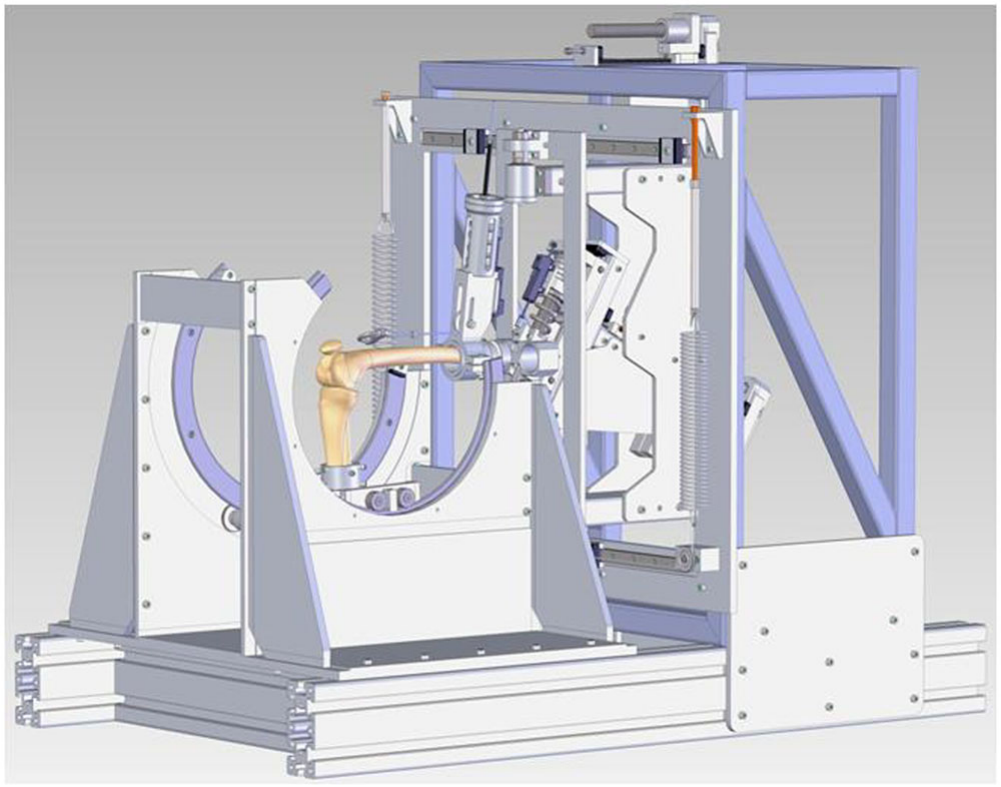
Drawing of the dynamic knee testing rig.

For each meniscal condition, the knee joint RoM was evaluated by measuring the angle during maximal passive flexion and extension, applied manually by the orthopaedic surgeon. Figure 4 shows that tantalum RSA beads were placed in the anterior horn, middle section and posterior horn of the meniscus prosthesis. Beads were also placed in the tibia, femur and in both horns and middle section of the native meniscus. Next, the cadaveric specimen was mounted in a dynamic knee testing apparatus specifically designed for this purpose (Figures 6 and 7), ensuring alignment between the center of rotation of the testing rig and the knee joint. Dedicated software was used to control knee flexion angle and joint load. Flexion and extension were applied by an electromotor rolling the tibial bone pot around the flexion/extension axis, while two spindle actuators could apply anteroposterior and proximodistal forces to the femoral bone pot. Varus/valgus, internal rotation and mediolateral translation were unrestricted at the femoral side.

After preconditioning the joint structures by five unloaded knee flexions, RSA measurements were performed at 0°, 15°, 30°, 60°, 90° and 120° of flexion. In addition, axial loads of 500 and 1000 N were applied at 0° and 15° flexion to assess meniscal extrusion under loading. Loads higher than 1000 N were not used to avoid fracture of the cadaveric specimens [16].

**Figure 8:**
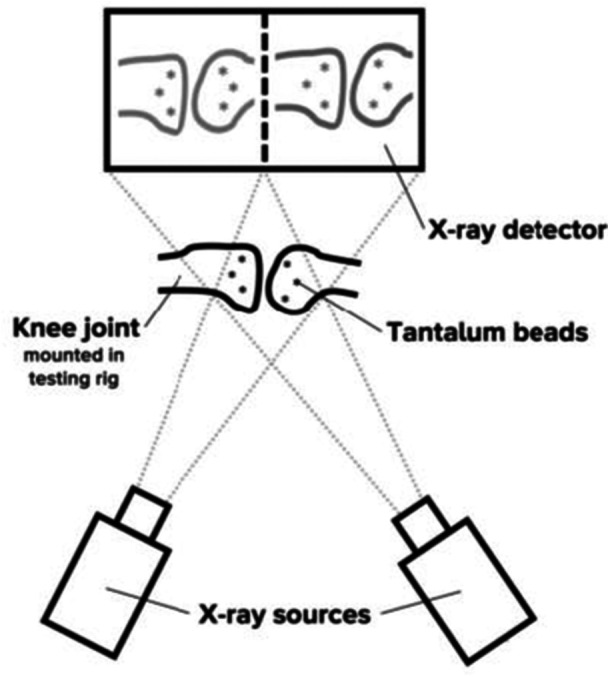
Schematic representation of the test setup for Roentgen stereophotogrammetric analysis (RSA).

**Figure 9:**
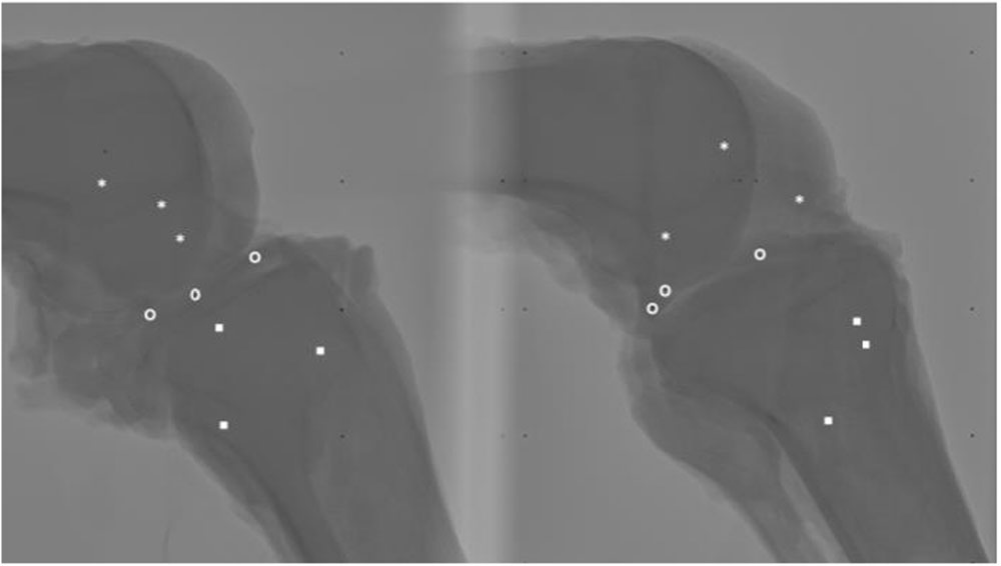
Example of a stereo Roentgen image, with beads detected in femur (*), meniscus (**o**) and tibia (■).

As shown in Figures 8 and 9, the test setup with two X‐ray sources (EcoRay, Seoul, South Korea) resulted in a stereo image on the detector (AGFA Healthcare, Mortsel, Belgium). From this image and an internally developed calibration system, the three‐dimensional position of the tantalum beads could be determined [27]. Considering tibia and femur as rigid bodies, translations of the femur and the different meniscus beads in the moving tibial reference frame were calculated relative to the position in full extension.

### Lachman test

**Figure 10:**
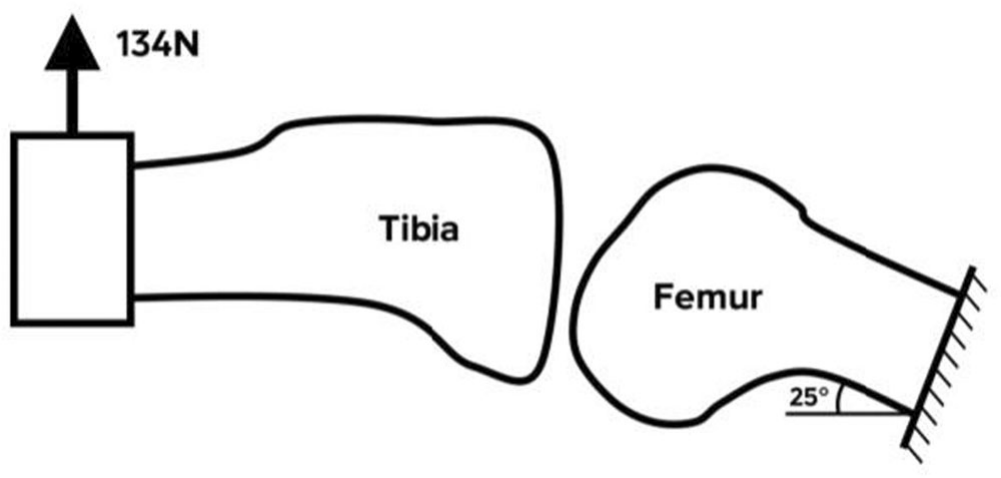
Schematic representation of the Lachman test.

A Lachman test can be used to diagnose ACL injuries, by evaluating passive anterior tibial translation in 20°–30° of flexion [7, 8]. A maximum anterior load of 134 N was applied during the test [2, 20]. In the current study, anterior knee joint laxity was investigated for the different meniscal conditions, with and without the intact ACL, by applying an anterior force to the tibia. As shown in Figure 10, the femur was mounted at an angle of 25°, while the tibia was horizontally attached to a hydraulic test device (MTS Systems, Eden Prairie, MN, USA).

Starting at the resting position of the intact knee joint, the tibia was moved upwards (anteriorly) at a rate of 5 mm/s, until a force of 134 N was reached and the corresponding displacement was recorded. As the native meniscus was previously resected, the combination of intact meniscus and resected ACL could not be evaluated.

### Statistical analysis

Given the exploratory nature of this study, it was decided to test six knees for evaluating the medial meniscal conditions and six more for the lateral side. Six knees would suffice to detect differences in translation of 3 mm, assuming a standard deviation of 1.6 mm and using a power of 80% [27]. One additional knee had to be included for the medial tests, as the Lachman test was unsuccessful in one of the tests.

The effects of different meniscal conditions on the defined outcome measures were studied using linear mixed models. Range of motion, femoral and meniscal kinematics during passive flexion, and anteroposterior tibial translation during the Lachman test were analysed separately. The cadaveric specimens were included in the model as random factors, while meniscal condition and flexion angle (if applicable) were included as fixed factors. No corrections were applied for multiple testing, as such adjustments can be overly conservative in small exploratory studies, increasing the risk of Type II errors and potentially obscuring meaningful trends. *p*‐Values below 0.05 were considered statistically significant. All statistical outcomes were calculated using R (version 4.4.1; R Foundation for Statistical Computing, Vienna, Austria).

## RESULTS

No significant differences in passive flexion or extension were found between the meniscus prostheses and the other meniscal conditions. Overall, the RoM was smallest for the native situation, which was obviously measured prior to all other meniscal conditions.

### Joint kinematics

Visually, no differences in joint movement were observed between the different meniscal conditions. In general, the RSA outcomes indicate comparable femoral translations for all conditions, although some differences were found. The complete set of femur translations can be found in the addendum.

**Figure 11:**
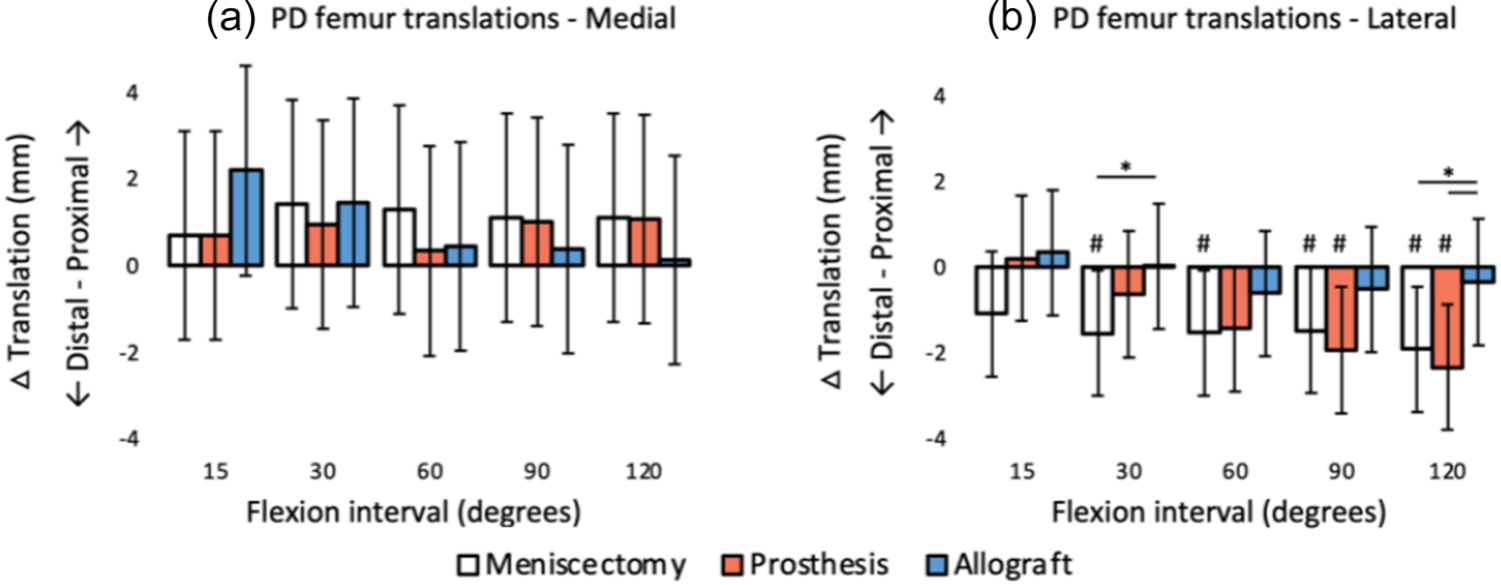
Effect on proximodistal femur translations during flexion, relative to the tibial plateau of different (a) medial meniscal conditions and (b) lateral meniscal conditions, relative to the intact native meniscus condition (mean difference and 95% confidence interval). *Significant difference (*p* < 0.05); ^#^Significant difference with the native meniscus (*p* < 0.05).

Figure 11 shows the difference in proximal femur translations between the native meniscus and the other meniscal conditions. Figure 11a demonstrates that implantation of the medial meniscus prosthesis had no significant effect on the proximodistal femur kinematics during flexion. Lateral meniscectomy and the lateral prosthesis resulted in a more distal femur position in multiple flexion angles, compared to the native condition.

#### Meniscus kinematics

**Figure 12:**
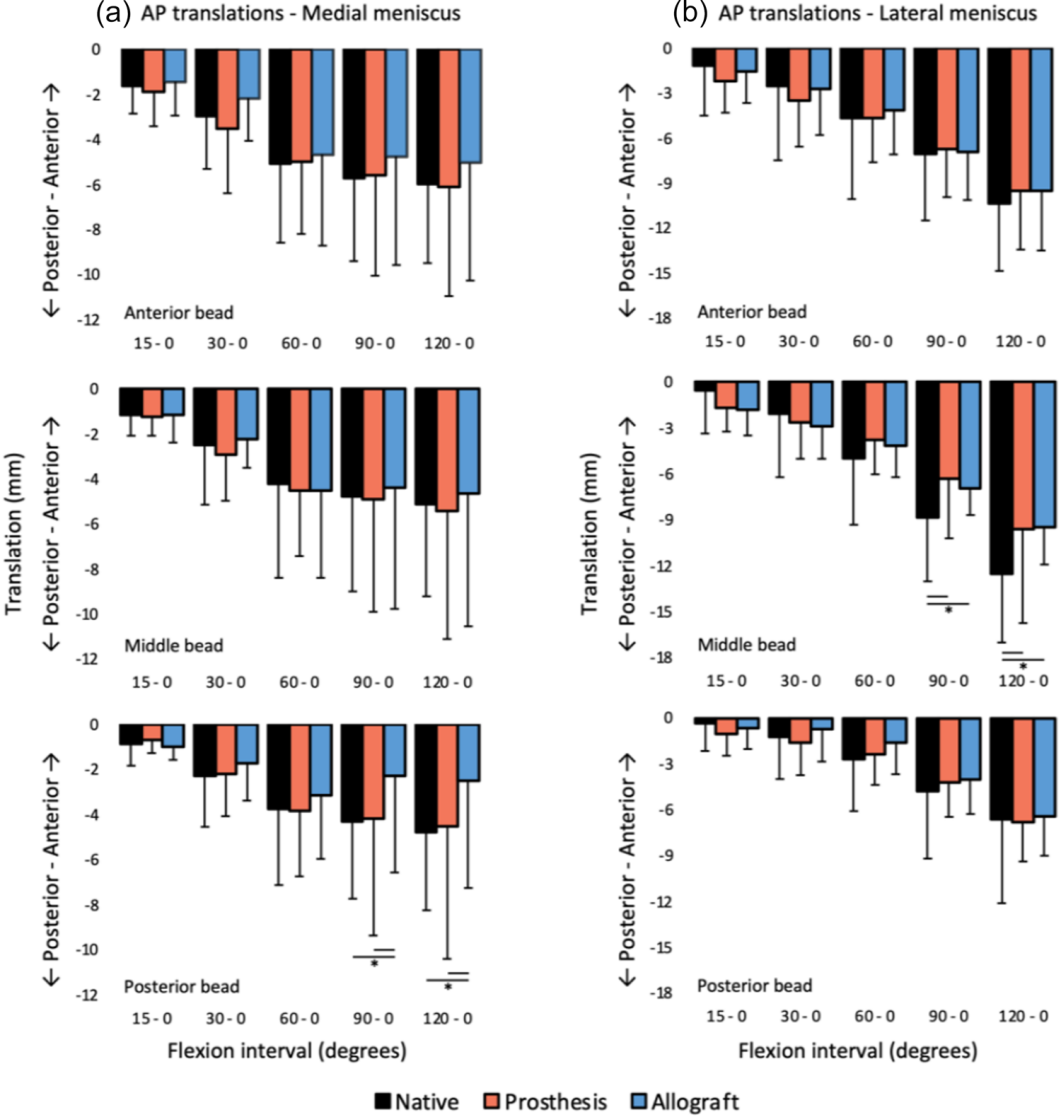
Anteroposterior translation of the different (a) medial and (b) lateral meniscus beads on the tibial plateau during passive flexion (mean ± standard deviation). Note that not all translation axes have the same scale. *Significant difference (*p* < 0.05).

The complete set of meniscal kinematics in different conditions can be found in the addendum. Figure 12 demonstrates that, during passive flexion, all beads of the medial meniscus prosthesis translate similarly to the corresponding beads in the native meniscus. The middle bead of the lateral prosthesis moved somewhat less than the native meniscus and behaved more comparable to the lateral meniscal allograft.

**Figure 13:**
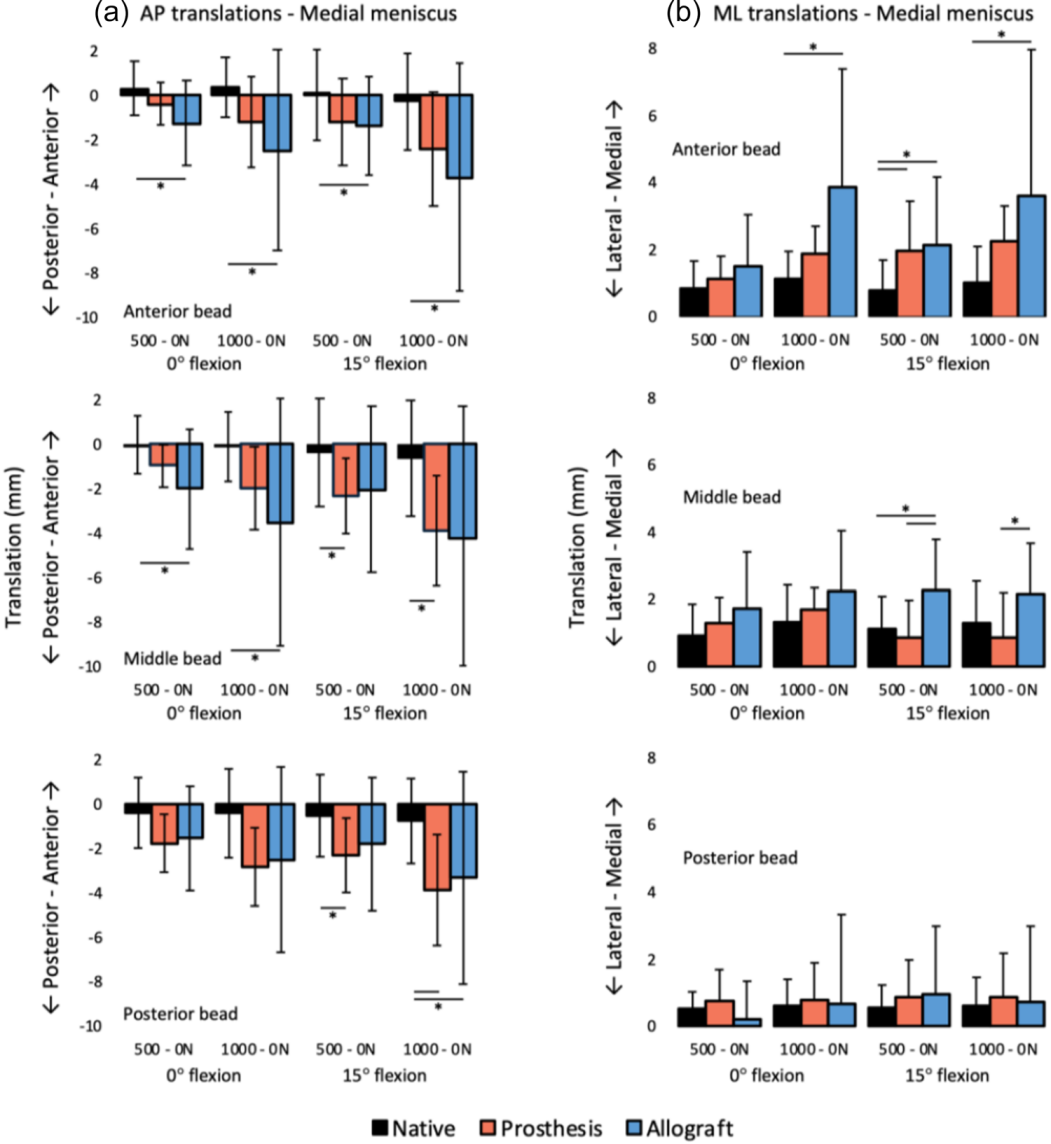
(a) Anteroposterior and (b) mediolateral translation of the different medial meniscus beads on the tibial plateau during axial loading (mean ± standard deviation). Note that not all translation axes have the same scale. *Significant difference (*p* < 0.05).

When loaded, the native medial meniscus beads hardly moved in the anteroposterior direction (Figure 13a). Both the medial prosthesis and medial allograft showed larger translations in this direction. Medial extrusion was similar between the native medial meniscus and medial prosthesis, although one significant difference was found (Figure 13b).

#### Lachman test

**Figure 14:**
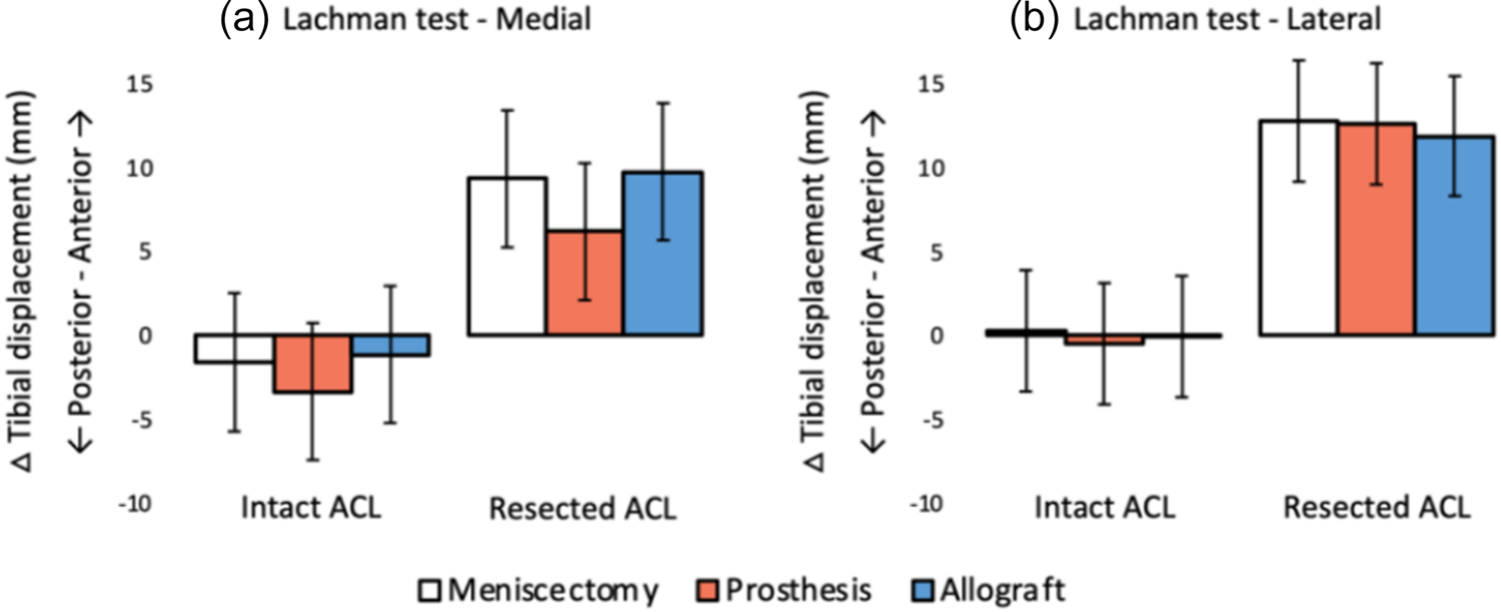
Effect on anterior tibial displacement of different (a) medial meniscal conditions and (b) lateral meniscal conditions, relative to the condition with intact native meniscus and intact anterior cruciate ligament (ACL) (mean difference and 95% confidence interval). All differences between intact ACL and resected ACL were significant (*p* < 0.05), all other differences were not statistically significant.

The results of the Lachman test, are shown in Figure 14, expressed as difference in laxity relative to the knee joint with intact ACL and intact native meniscus. The differences in anterior tibial displacement between intact and resected ACL were statistically significant, for all medial and lateral meniscal conditions. None of the differences within the same ACL condition were significant.

## DISCUSSION

The main finding of this study was that medial and lateral meniscus prostheses do not adversely affect knee joint RoM and kinematics, as they are able to move similarly to the native meniscus. In addition, relatively low axial forces were able to extrude the prostheses at least as much as the native meniscus, confirming the increased in‐plane flexibility of the prosthesis and its fixation. These results are promising, as the previous medial prosthesis design led to limited RoM and high loads on the prosthesis, resulting in unsatisfactory clinical results and prosthesis breakage [23]. No significant effects on knee joint laxity were found in a simulated Lachman test.

### Joint kinematics

It was expected that none of the meniscal conditions would influence knee joint RoM or kinematics. Nonetheless, some unanticipated findings were reported. First, the measured RoM was always smallest for the native meniscus condition which was always tested first. This finding probably indicates an increased laxity of the knee joint during testing, caused by deterioration of the cadaveric tissue and multiple procedures required to apply the different meniscal conditions. This effect was limited between the other meniscal conditions, due to the alternation in test sequence, which could not be implemented for the native condition.

Although not observed visually, some significant differences in joint kinematics were found between the native meniscus and the other meniscal conditions, such as the prosthesis. These differences are difficult to explain by characteristics of the different menisci, as they demonstrate comparable translations over the tibial plateau. A probable reason for these differences, and the large variation in general, is the initial position. Although carefully marked, the initial position for each meniscal condition had to be determined manually, making the kinematic analysis prone to errors.

### Meniscus kinematics

Although different methods have been described to assess meniscus kinematics during flexion, the overall outcomes of this study correspond to literature [21]. The magnitude of the measured prosthesis translations is in the range of a previous study including a predecessor of the current medial meniscus prosthesis [26]. Small differences in meniscal behaviour are to be expected due to differences in geometry and mechanical properties. The meniscus prosthesis aims to mimic the native meniscus but is somewhat stiffer, especially in the compressive direction. This effect is enhanced by the fact that the test was not performed in physiological conditions, as a warm and wet environment would have resulted in a more flexible PCU [10]. On the other hand, tape fixation of the prosthesis horns allow for more translation than the rigid tibial attachment of the native meniscus. Both the meniscal body and suture fixation of MAT are flexible, overall resulting in the largest meniscal translations.

### Lachman test

Resection of the ACL resulted in an increased anteroposterior laxity of approximately 10 mm, corresponding to the highest IKDC classification. Implantation of the medial meniscus prosthesis reduced this effect by approximately one third, although statistical significance could not be demonstrated. Since it is known that the native medial meniscus has a stabilising function under tibial AP load, a similar attribute for the medial prosthesis is plausible [12]. This study did confirm that the medial and lateral prostheses do not have any negative effects on knee joint laxity.

### Limitations

Besides always testing the native meniscus first, one additional limitation for the determination of knee joint RoM was identified. Passive flexion and extension were measured at a force applied manually, mimicking clinical practice. Differences in applied force could have led to some variation in resulting RoM, although no clear indication for this effect was found in the results.

Limitations of the kinematic study also include the sub‐physiological loads of 500 and 1000 N, where activities of daily living may result in knee loads of up to 5000 N [4]. The age of the knees used led to a decreased bone quality and a limitation on the applied load, especially given the fact that multiple conditions had to be evaluated in multiple tests. However, the differences found between 500 and 1000 N were relatively small, indicating that the effect of further increasing the axial load would not lead to completely different results. This assumption is supported by an earlier biomechanical study, which demonstrates that, when increasing loads, a larger portion of the forces is transferred through the direct tibiofemoral cartilage contact rather than putting more strain on the meniscus or the prosthesis [16].

Some unexpectedly large differences in kinematics were found, especially for the meniscal allografts. It was found that one outlying result could already lead to statistically significant differences. As in most cases no specific cause, such as a dislocated bead, could be identified, removing these outliers was not justified. Choosing a larger sample size may have reduced the effect of outliers. The small sample size is therefore probably the most important limitation of this study, especially when it comes to statistical significance of the found differences. Future clinical studies should confirm that the medial and lateral meniscus prostheses have no adverse effect on RoM and joint pain.

The results of the Lachman test could only be used when comparing with the intact knee joint, as the absolute AP translations were excessive due to bending effects of the long bones. Nonetheless, the found results were in the same order of magnitude for the different cadaveric specimens. The large standard deviation and a decrease in average laxity after meniscectomy may be at least partially caused by positioning effects. Although the initial anteroposterior position was standardised, the initial proximodistal position was not constrained by the test setup and has a potential effect on the measured anteroposterior laxity.

Unfortunately, the Lachman test could not be performed with the intact native meniscus and a resected ACL, as the native meniscus had to be removed during the kinematic study with different meniscal conditions. This would have been a valuable addition, especially to validate the experimental design by comparing with available literature.

## CONCLUSIONS

The artificial medial and lateral meniscus prostheses did not affect knee range of motion. Only minor differences in knee joint and meniscus kinematics were found between the prostheses and the native meniscus, and no effect on joint laxity was found. Future clinical studies should confirm that the medial and lateral meniscus prostheses have no adverse effect on joint kinematics and result in favourable outcomes for patients with post‐meniscectomy pain.

## AUTHOR CONTRIBUTIONS

Branco S. van Minnen participated in the design of the study, execution of the mechanical tests, processing of results and drafting the manuscript. Alexis R. Sturm participated in execution of the mechanical tests, processing of results and drafting the manuscript. Albert J. van der Veen participated in the design of the study, specimen preparation and execution of the mechanical tests. Sebastiaan A.W. van de Groes participated in the design of the study and helped to interpret the data from a clinical point of view. Nico Verdonschot participated in the design of the study and helped to interpret the outcomes from a biomechanical point of view. Tony G. van Tienen participated in the design of the study, performed surgical procedures and helped to interpret the outcomes. All authors read, reviewed and approved the final manuscript.

## CONFLICT OF INTEREST STATEMENT

Branco van Minnen and Albert van der Veen are employed at ATRO Medical B.V. Tony van Tienen receives a management fee from ATRO Medical B.V. Branco van Minnen, Albert van der Veen and Tony van Tienen own stocks in ATRO Medical B.V. Branco van Minnen, Albert van der Veen and Tony van Tienen are inventors on applicable patents, but have no ownership.

## ETHICS STATEMENT

Approval for the use of cadaveric material was obtained from the anatomy departments of the Radboud University Medical Center (Nijmegen, The Netherlands) and the VU University Medical Center (Amsterdam, The Netherlands).

## Figures and Tables

**Figure 15:**
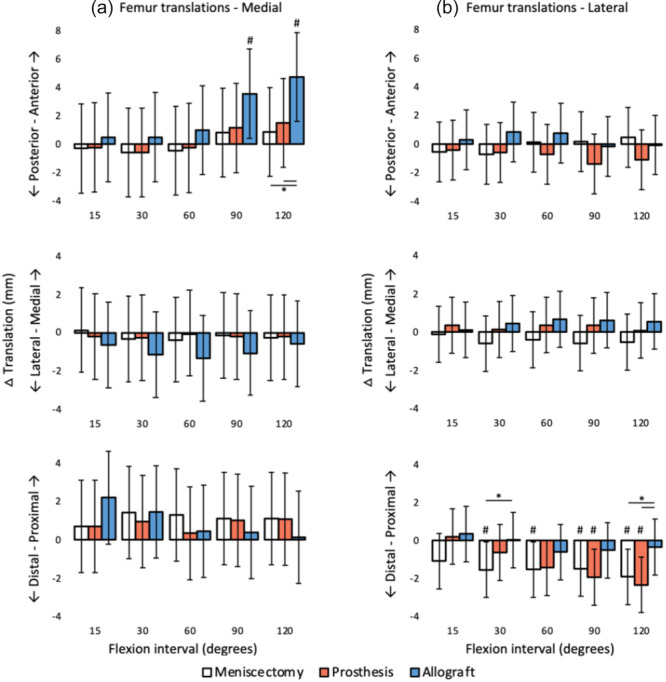
Effect on femur translations during flexion, relative to the tibial plateau of different (a) medial meniscal conditions and (b) lateral meniscal conditions, relative to the intact native meniscus condition (mean difference and 95% confidence interval). Note that not all translation axes have the same scale. *Significant difference (*p* < 0.05); ^#^Significant difference with the native meniscus (*p* < 0.05).

**Figure 16:**
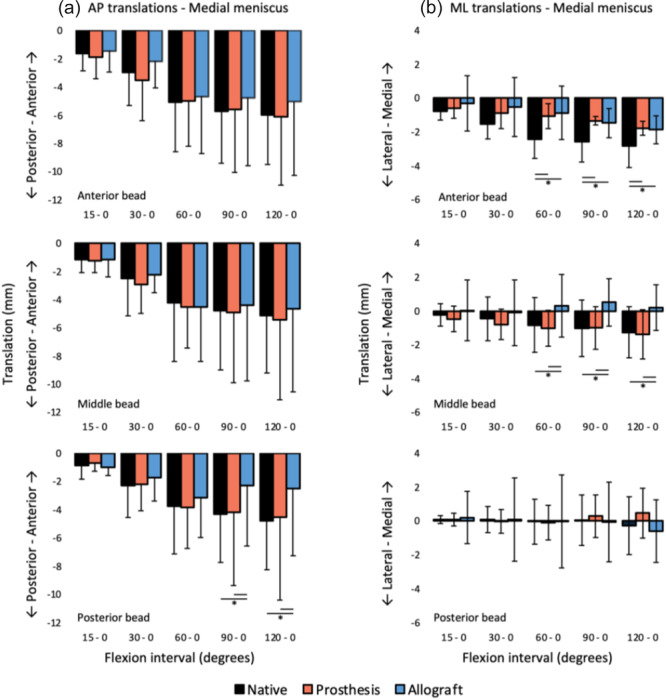
(a) Anteroposterior and (b) mediolateral translation of the different medial meniscus beads on the tibial plateau during passive flexion (mean ± standard deviation). Note that not all translation axes have the same scale. *Significant difference (*p* < 0.05).

**Figure 17:**
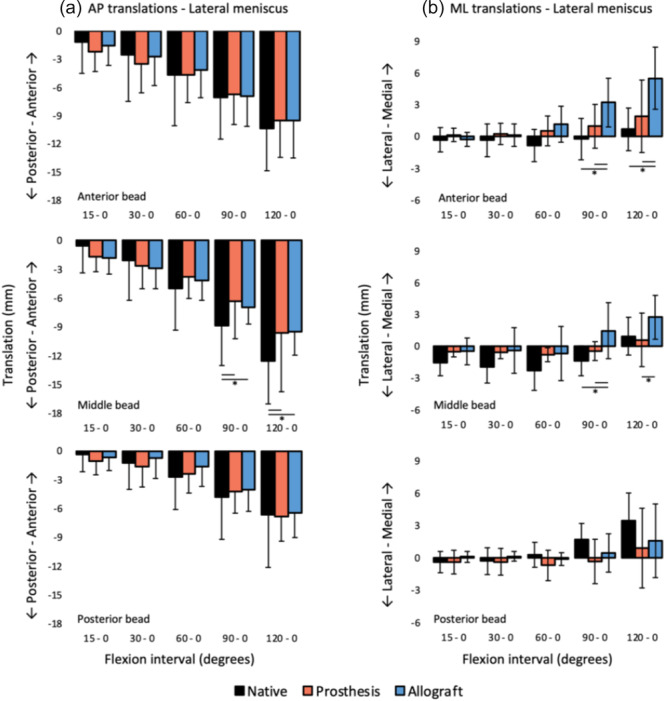
(a) Anteroposterior and (b) mediolateral translation of the different lateral meniscus beads on the tibial plateau during passive flexion (mean ± standard deviation). Note that not all translation axes have the same scale. *Significant difference (*p* < 0.05).

**Figure 18:**
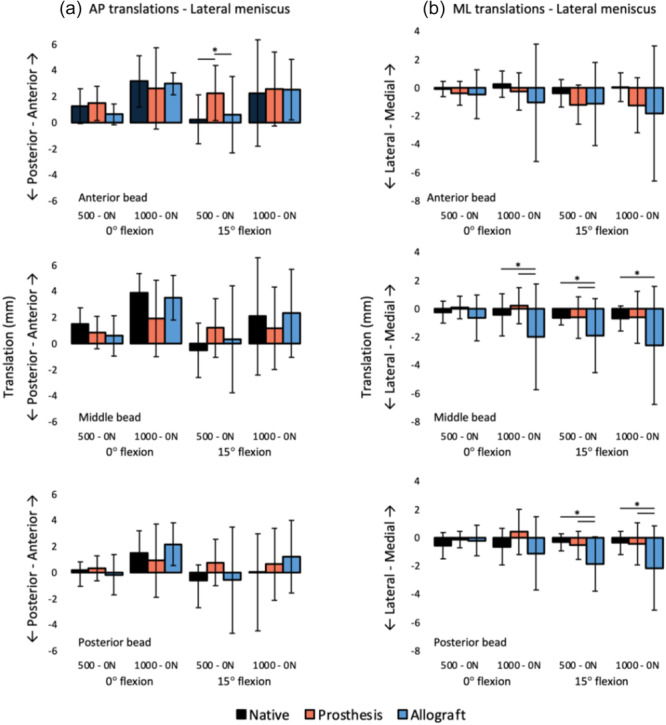
(a) Anteroposterior and (b) mediolateral translation of the different medial meniscus beads on the tibial plateau during axial loading (mean ± standard deviation). Note that not all translation axes have the same scale. *Significant difference (*p* < 0.05).

## Data Availability

The data set supporting the conclusions of this article will be made available after publication of the article.

## – Complete set of measured translations

Figures 15, 16, 17, 18 contain all measured translations of femur and meniscus, and supplement the outcomes summarised in Figures 11, 12, 13, 14.
